# Pediatric asthma comprises different phenotypic clusters with unique nasal microbiotas

**DOI:** 10.1186/s40168-018-0564-7

**Published:** 2018-10-04

**Authors:** Marcos Pérez-Losada, Kayla J Authelet, Claire E Hoptay, Christine Kwak, Keith A Crandall, Robert J Freishtat

**Affiliations:** 1 Computational Biology Institute, Milken Institute School of Public Health,, George Washington University, Innovation Hall, Suite 305, 45085 University Drive, Ashburn, VA 20147 USA; 2 Department of Epidemiology and Biostatistics, Milken Institute School of Public Health, George Washington University, Washington, DC, 20052 USA; 3 CIBIO-InBIO, Centro de Investigação em Biodiversidade e Recursos Genéticos, Universidade do Porto, Campus Agrário de Vairão, 4485-661 Vairão, Portugal; 4 Division of Emergency Medicine, Children’s National Medical Center, Washington, DC, 20010 USA

**Keywords:** 16S rRNA, Asthma, Microbiome, Nose, Phenotype

## Abstract

**Background:**

Pediatric asthma is the most common chronic childhood disease in the USA, currently affecting ~ 7 million children. This heterogeneous syndrome is thought to encompass various disease phenotypes of clinically observable characteristics, which can be statistically identified by applying clustering approaches to patient clinical information. Extensive evidence has shown that the airway microbiome impacts both clinical heterogeneity and pathogenesis in pediatric asthma. Yet, so far, airway microbiotas have been consistently neglected in the study of asthma phenotypes. Here, we couple extensive clinical information with 16S rRNA high-throughput sequencing to characterize the microbiota of the nasal cavity in 163 children and adolescents clustered into different asthma phenotypes.

**Results:**

Our clustering analyses identified three statistically distinct phenotypes of pediatric asthma. Four core OTUs of the pathogenic genera *Moraxella*, *Staphylococcus*, *Streptococcus*, and *Haemophilus* were present in at least 95% of the studied nasal microbiotas. Phyla (Proteobacteria, Actinobacteria, and Bacteroidetes) and genera (*Moraxella*, *Corynebacterium*, *Dolosigranulum*, and *Prevotella*) abundances, community composition, and structure varied significantly (0.05 < *P* ≤ 0.0001) across asthma phenotypes and one of the clinical variables (preterm birth). Similarly, microbial networks of co-occurrence of bacterial genera revealed different bacterial associations across asthma phenotypes.

**Conclusions:**

This study shows that children and adolescents with different clinical characteristics of asthma also show different nasal bacterial profiles, which is indicative of different phenotypes of the disease. Our work also shows how clinical and microbial information could be integrated to validate and refine asthma classification systems and develop biomarkers of disease.

**Electronic supplementary material:**

The online version of this article (10.1186/s40168-018-0564-7) contains supplementary material, which is available to authorized users.

## Background

Pediatric asthma is the most common chronic childhood disease and a major public health problem in the USA, currently affecting 9.3% of the children (~ 7.0 million), and prevalence continues to rise [1–3]. Prevalence is particularly high (13.9%) in Washington, DC, mainly among African Americans [2], who nonetheless still represent one of the least studied ethnicities with regards to asthma [4]. Childhood asthma is the third leading cause of hospitalization (137,000 cases) among US children, accounting annually for 640,000 emergency department visits [5, 6]. It is also a major cause of school absenteeism (~ 14.4 million lost school days/year) [7], and treatment cost is estimated at $3.2 billion/year [8, 9].

Pediatric asthma is recognized as a complex condition with differences in severity, natural history, comorbidities, and treatment response [10–13]. A longstanding debate is whether pediatric asthma (and asthma in general) is a single disease with a variable presentation or several diseases that have variable airflow obstruction as a common feature. Wenzel [14] proposed that the different phenotypes expressed by patients with asthma are partly dependent on different disease processes in each individual. Thus, the diagnostic label “asthma” likely encompasses many different disease variants with different etiologies and pathophysiologies [10, 11, 15]. Typically, a child’s asthma is described in terms of disease phenotypes, which summarizes observable characteristics (clinical, physiological, morphologic, and biochemical), as well as the response to different treatments; thus, they are clinically relevant in terms of presentation, triggers, and treatment response. Asthma phenotypes have long been described by clinicians from their own practice experience, but now are better characterized by applying clustering approaches to patient clinical information collected from cohorts of asthmatic patients (i.e., asthma phenotypic clusters) [16–19]. Using hierarchical clustering, for example, our group has distinguished in a previous study three phenotypic clusters of pediatric asthma in a cohort of children and adolescents from the Washington, DC area (The AsthMaP Project) [18].

The application of novel culture-independent techniques of Next-Generation Sequencing (NGS) has already demonstrated that bacterial communities living in the respiratory airways play a significant role in the onset, development, and severity of asthma [20–29]. Moreover, microbiome research has also shown that the nose is a major reservoir for opportunistic pathogens [30, 31], which can from there spread to other sections of the respiratory tract and potentially cause asthma, but also otitis media or pneumonia, or invade the bloodstream to cause sepsis and meningitis [27, 28, 32–36].

The relationship between airway microbiota and asthma phenotypes is still poorly understood [37–39]. A few studies have explored the interaction in adults [37, 38] and have shown significant variation in microbial diversity and the abundance of pathogenic taxa across asthma phenotypes. Whether differences in the composition of microbial populations (pathogenic and commensal) could contribute to asthma in children and adolescents remains to be determined. Defining the relationships between pediatric asthma phenotypes and nasal airway microbiota could ultimately inform our understanding of asthma pathophysiology and could help identify prognostic markers [18, 37].

Here, we first applied cluster analysis to clinical, physiological, and biochemical information collected from 163 children and adolescents from Washington, DC belonging to a new cohort (AsthMaP-2) to define phenotypes of pediatric asthma. Then we generated 16S rRNA microbial profiles for those same patients (i) to assess if phenotypic clusters of asthma were associated with nasal bacterial diversity, (ii) to identify bacterial taxa that discriminate among asthma phenotypes, and (iii) to determine the contribution of clinical characteristics to variation in the composition and structure of the nasal microbiome.

## Methods

### Cohort

AsthMaP-2 is an ongoing study of urban children and adolescents, mainly African Americans (81% of the subjects in this study with Whites and undetermined accounted for 2% and 17%, respectively), designed to find associations among airway microbes, environmental exposures, allergic sensitivities, genetics, and asthma. AsthMaP-2 represents a unique sample of otherwise healthy children recruited from the metropolitan Washington, DC area with physician-diagnosed asthma present for at least 1 year. Subjects were enrolled from all the sites in our citywide pediatric and adolescent health system. They were evaluated in our Clinical Research Center via parental interviews, aeroallergen skin testing, nasal sampling, and blood collection. Participants were enrolled between 2013 and 2015 at one study visit at least 4 weeks after completion of their most recent oral steroid dose (baseline) and then followed for 1 year. Individuals who reported a medical history of chronic or complex cardiorespiratory disease were ineligible.

### Sample collection

A total of 205 nasal washes were collected from 163 children and adolescents (ages 6 to 18 years) enrolled in the AsthMaP-2 study at Children’s National Medical Center (Washington, DC). Forty-two of those patients came back for an additional visit (5.5 to 6.5 months apart) and one additional sample (E3) was taken (Additional file 1: Table S1). Washes were procured by instilling 5 ml of isotonic sterile saline buffer into each nare, holding it for 10 s and then blowing into a specimen collection container. Washes were kept in ice while being collected and then stored at − 80 °C until needed.

### High-throughput sequencing

Total DNA was extracted using the QIAGEN QIAamp DNA Kit (Catalog # 51304). Before adding the ATL buffer, samples were pre-incubated in 100 uL of lysozyme-TE buffer pH = 8.0 for 30 min at 37 °C. All extractions yielding > 2 ng/μ of total DNA, as indicated by NanoDrop 2000 UV-Vis Spectrophotometer measuring. DNA extractions were prepared for sequencing using the Schloss’ MiSeq_WetLab_SOP protocol (09.2015) in Kozich et al. [40]. Each DNA sample was amplified for the V4 region (~ 250 bp) of the 16S rRNA gene and libraries were sequenced in a single run of the Illumina MiSeq sequencing platform at the University of Michigan Medical School. Negative controls processed as above showed no PCR band on an agarose gel.

### Phenotype cluster analysis

A total of 163 children and adolescents from the AsthMaP-2 cohort were included in the cluster analysis. We collected information for 29 clinical variables (Additional file 1: Table S1) during their first visit—no new clinical data were collected during the second visit. Those 29 variables were (in alphabetical order) ACT (Asthma Control Test) score, age (years), age of onset of asthma symptoms (years), allergic rhinitis, antihistamine use in the last 2 weeks, beta agonist use in the last 2 weeks, blood eosinophils (%), BMI (body mass index) percentile, cold in the last 4 weeks, eczema, FEF (forced expiratory flow), FEV_1_ (forced expiratory volume in one second) change with bronchodilator, FEV_1_/FVC (functional vital capacity), hospital visit for sample collection, inhaled steroid use in the last 2 weeks, ITG (Integrated Therapeutics Group’s Child Asthma Short Form) composite score (see Table 1), leukotriene modifier use in the last 2 weeks, meteorological season during sample collection, NAEPP (National Asthma Education and Prevention Program) severity classification, pets, positive on skin allergen test, postFEV1, preFEV1, preterm birth (< 35 weeks of gestation), race, respiratory infection in the last 4 weeks, sex, total serum IgE (IU/ml), and vitamin D use in the last 2 weeks. The ACT is a patient-driven test developed to identify those with poorly controlled or refractory asthma. Poor ACT scores indicate the patient’s symptoms persist despite the use of conventional treatments. The ITG indicates the asthma-related quality of life and is a self-reported functional health questionnaire. It is scaled between 0 and 100. All collected variables were standardized in binary fashion for categorical variables or using a *z* score for continuous variables. As in previous asthma studies [16, 18], relevant variables were chosen if they are measured in the clinical evaluation of asthma and describe asthma phenotypes. Additionally, selection of multiple variables representing the same aspect of asthma was avoided. Principal components analysis (PCA) based on Euclidean distances was then carried out on the eleven selected variables in Table 1 to identify key clinical components relevant to asthma diagnosis and assessment. Nine of these 11 asthma-relevant clinical variables were also used in our previous phenotype cluster analysis [18] of the AsthMaP cohort—a different cohort from that studied here.

**Table 1:** Varimax rotation of 11 asthma-relevant variables

Variable	Component			
	1	2	3	4
ACT score^a^	0.798			
ITGc—composite score^a^	0.984			
ITGf—functional limitations score	0.864			
ITGd—daytime symptoms score	0.848			
ITGn—nighttime symptoms score	0.842			
Post-bronchodilator FEV_1_ (% predicted)				0.967
Post-bronchodilator FEV_1_/FVC (% predicted)			0.765	
FEV_1_ change with bronchodilator			0.784	
Age, years^a^		− 0.551		
Blood eosinophil, %^a^		0.761		
Total serum IgE, IU/mL		0.741		

Extraction method: principal component analysis. Rotation method: varimax with Kaiser normalization. Bartlett’s test of sphericity < 0.001. KMO measure of sampling adequacy: 0.471. *ACT* asthma control test, *ITG* Integrated Therapeutics Group’s Child Asthma Short Form, *FEV* forced expiratory volume, *FEF* forced expiratory flow, *IgE* immunoglobulin E. ^a^Variable used in cluster analysis.

Principal component factors were identified using a varimax rotation of the eleven variables. As in Benton et al. [18], cluster analysis was performed in two stages using variables representative of the principal components. In the first stage, hierarchical clustering of the variables using between-groups linkage yielded the probable number of clusters present in AsthMaP-2. A k-means cluster analysis was then performed using this estimated number of clusters. This stage was repeated while specifying one more or one less cluster than the estimate to ensure that the most representative model was obtained. Additionally, the k-means cluster analysis was repeated several times within random AsthMaP-2 subpopulations to ensure reproducibility. Differences between clusters were derived using one-way analysis of variance for normally distributed continuous variables, Kruskal-Wallis for nonparametric continuous variables, and chi-square tests for categorical variables. All statistical tests were performed with SPSS Statistics 17.0 (SPSS, Chicago, IL).

### Microbiome analyses

Raw FASTQ files were processed in mothur v1.35.1 [41] as indicated in the MiSeq SOP (www.mothur.org/wiki/MiSeq_SOP). Default settings were used to minimize sequencing errors [42]. We removed any sequences with ambiguous bases (maxambig = 0). We sequenced both negative controls and mock communities (reference samples with a known composition) to detect contaminating microbial DNA within reagents and measure sequencing error rate. We did not find evidence of contamination and our sequencing error rate was as low as 0.0071%. Clean paired-end sequences were joined into contigs of ~ 250 bp and then aligned to the SILVA128-based bacterial reference alignment at www.mothur.org. Contigs > 275 bp were removed. Chimeras were also removed using uchime [43], and non-chimeric sequences were classified using a naïve Bayesian classifier [44]. Sequences were clustered into Operational Taxonomic Units (OTUs) at the 0.03 similarity threshold (species level). A consensus taxonomy was generated based on the classification of sequences clustered within an OTU. OTU sequence representatives and taxonomy were then converted to a BIOM file for subsequent analyses and all OTU singletons (*n* = 1) were eliminated. We normalized our samples using the negative binomial distribution as recommended by McMurdie and Holmes [45] and implemented in the Bioconductor package DESeq2 [46]. This approach simultaneously accounts for library size differences and biological variability. Microbial normalized counts generated this way are referred to as taxon abundances throughout the text. Trees for phylogenetic diversity calculations were constructed using FastTree and midpoint rooting [47]. Taxonomic alpha-diversity was estimated using Shannon and ACE indices, while phylogenetic alpha-diversity was calculated by the Faith’s phylogenetic diversity index [48]. Beta-diversity was estimated using phylogenetic UniFrac (unweighted and weighted), Bray-Curtis, and Jaccard distances. The dissimilarity between samples was explored using principal coordinates analysis (PCoA). We also carried out a Mantel correlation test and a Procrustes [49] analysis comparing clinical diversity (29 variables) across 163 patients with their microbiome 16S rRNA profiles on the basis of Euclidean similarity metrics and using 10,000 permutations for each test.

We used linear mixed-effects (LME) models analysis, as implemented in the lmer4 R package [50], to investigate associations between alpha-diversity indices and taxa (genera and phyla) abundances (response) and asthma phenotypes (predictor), while accounting for non-independence of subjects (random effect). We have included random effects in our LME models to account for the fact of that 42 patients were sampled twice during the study. We also investigated the potential contribution of other clinical characteristics of the AsthMaP-2 cohort to variation in the composition of the microbiome. Hence, 23 out of the 29 variables listed above were included in the initial LME analyses. To avoid redundancy, we did not include the six variables (ACT score, age, BMI percentile, ITG composite score, percentage of blood eosinophils, and sex) used in the phenotype cluster analysis (see the “Results” section below) to identify asthma phenotypes (composite variable). We also tested LME models with random intercepts and random slopes and different orders of factors. Initial LME models including the 23 variables listed above were compared using the function lmerTest, which performs automatic backward elimination of factors. ANOVA type III tests with Satterthwaite approximation for degrees of freedom were also carried out for hypothesis testing. Model assumptions in final LME models were validated using residual versus fit plots and a normal probability plots.

Beta-diversity UniFrac indices were compared using permutational multivariate analysis of variance (adonis) as implemented in the vegan R package [51]. Adonis models were compared using the Akaike Index Criterion [52]. Significance was determined through 10,000 permutations.

We believe that only statistically relevant factors in the dataset under study should be included in the final LME models to avoid subjectivity of choice and over-parametrization. Our preliminary LME and adonis analyses showed that random slopes did not have a significant impact on any representation of microbial diversity or taxon abundance. Three co-variables (vitamin D use, antihistamine use, and leukotriene modifier use), however, were significantly associated with one microbial diversity index or one taxon abundance in our LME analyses, but none of those tests were significant for the variable of interest (asthma phenotype). Similarly, preterm birth was significantly associated (*P* < 0.025) to several diversity indices and taxon abundances. Hence, our final (most parsimonious) LME and adonis models included one predictor (asthma phenotype) and one co-variable (preterm birth). We found no significant interactions between asthma phenotype and preterm birth.

Differences in microbial abundances for phyla and genera were also estimated using the Wald test with Cook’s distance correction for outliers (DESeq2 package) while accounting for preterm birth. We applied the Benjamini-Hochberg method at alpha = 0.05 to correct for multiple hypotheses testing [53, 54]. All the analyses above were performed in mothur, QIIME [55], R [56], and RStudio [57].

Microbial organisms coexist in complex ecological networks with various symbiotic relationships. Thus, the presence or abundance of certain individual bacteria most likely affects the presence of others because of those ecologic interactions [58]. We, therefore, estimated networks of co-occurrence of bacterial genera to assess similarities and differences among microbial communities in children and adolescents with different asthma phenotypes. Networks were built in MEGAN [59] using the following parameter settings: threshold = 0.01% (minimum count required for a taxon to be considered present in a sample), minimum prevalence = 10% and maximum prevalence = 100% (minimum and maximum percentage of samples in which a taxon can occur, respectively), and probability = 0.9 and 0.95 (minimum probability that a co-occurrence between two taxa A and B must attain so as to be represented by an edge in the graph).

## Results

### The AsthMaP-2 cohort is comprised of phenotypic clusters

#### Principal component analysis

Eleven key clinical variables relevant to asthma diagnosis and assessment in AsthMaP-2 were selected for PCA (Table 1). Varimax rotation of those variables identified four principal components representing symptoms/impairment, airway reactivity, mucosal evidence of allergy, and systemic evidence of allergy (Table 1). The analysis converged in six iterations. About 70% of the total variance was explained by these four components, while each of them accounted for 28.4%, 19.4%, 12.4%, and 11.3% of the variance after rotation sums of squared loadings. Four highly informative variables were selected for cluster analysis: ACT, ITGc, age, and blood eosinophil (* in Table 1). Two additional variables (sex and BMI percentile) known to be important factors in asthma phenotype were also included in the cluster analysis. Additionally, the most informative of the 29 variables representing asthma status and asthma-related quality of life were compared among clusters (Table 2). It should be noted that *P*-values for ACT, ITGc, age, blood eosinophil, sex, and BMI percentile are not true tests of variance since they were used to maximize distances between the clusters.

**Table 2:** Comparison of asthma characteristics in overall cohort and among asthma phenotypic clusters (APC)

Variable	All (*n* = 163)	APC1 (*n* = 51)	APC2 (*n* = 63)	APC3 (*n* = 49)	*P* value
Sex, % male^a^	52.6	29.5	65.1	63.2	< 0.001
Age, years (SE)^a^	11.0 (0.3)	12.7 (0.5)	9.1 (0.3)	11.6 (0.5)	< 0.001
Age of onset of asthma symptoms, years (SE)	4.1 (0.2)	4.9 (0.5)	3.4 (0.3)	4.3 (0.5)	0.0046
BMI percentile (SE)^a^	72 (2.2)	81.3 (3.0)	81.1 (2.7)	53.7 (4.5)	< 0.001
Pre-bronchodilator FEV_1_, % predicted (SE)	85.4 (1.4)	88 (2.0)	84.8 (2.6)	83.2 (2.6)	0.401
FEV_1_ change with bronchodilator (SE)	5.8 (0.4)	10.3 (1.6)	8.6 (1.6)	14.4 (4.4)	0.299
Post-bronchodilator FEV_1_, % predicted (SE)	99.2 (6.0)	96 (2.2)	93.6 (2.9)	109.2 (18.6)	0.518
NAEPP Severity	3 (2, 4)	3 (2, 4)	3 (2, 4)	3 (2, 4)	0.3
ACT score^a^	20.4 (0.3)	16.2 (0.5)	21.6 (0.3)	23.1 (0.4)	< 0.001
ITGc—composite score (IQR)^a^	68.9 (54.2, 86.5)	51.2 (41.7, 57.3)	71.3 (64.1, 79.2)	84.2 (72.9, 95.8)	< 0.001
Blood eosinophil, % (IQR)^a^	5.8 (2.25, 5.75)	4.5 (1.8, 6.3)	8.9 (5.3, 11.7)	3.2 (1.2, 4.7)	< 0.001
Total serum IgE (IQR)	545 (99, 710)	579 (97, 751)	675 (139, 912)	330 (75, 368)	0.013
Positive skin allergen tests, %	73	35	46	25	0.006
Beta agonist use, %	27	11	8	11	0.471
Inhaled steroid use, %	54.6	17.2	23.9	13.5	0.342

*FEV* forced expiratory volume, *FEF* forced expiratory flow, *NAEPP* National Asthma Education and Prevention Program, *ACT* asthma control test, *ITG* Integrated Therapeutics Group’s Child Asthma Short Form, *IgE* immunoglobulin E, *SE* standard error, *IQR* interquartile range. ^a^Variable used in cluster analysis

#### Cluster analysis

The cluster of 163 children and adolescents from the AsthMaP-2 cohort using six asthma-relevant clinical variables resulted in a four-cluster best-fit model with distinct asthma phenotypes. One cluster, however, contained only one single individual with mild asthma and was, therefore, eliminated because its microbiome diversity and composition cannot be statistically compared to other larger clusters. The three remaining clusters differed in their clinical characteristics (Additional file 2: Figure S1 and Table 2). Asthma phenotypic cluster 1 (APC1) was predominantly female (69.5%) with a lower mean ACT score [mean (SE)] [16.2 (0.5)] and ITGc score [mean (IQR)] [51.2 (41.7, 57.3)] (Table 2). APC1 also had the latest mean age of onset of asthma of 4.9 (0.5) years and the highest BMI percentile [81.3 (3.0)] but was not statistically different from APC3 and was within the standard error of APC2. One or more positive allergen skin test (%) was significantly different between the three groups (*P* = 0.006).

Asthma phenotypic cluster 2 (APC2) had the highest positive allergen tests at 46%, with APC1 and APC3 having 35% and 25%, respectively (Table 2). APC2 also had the highest blood eosinophil % [mean (IQR)] [8.9 (5.3, 11.7)] and highest serum IgE value [674.9 (139, 912)]. Serum IgE was also statistically significantly different between the three phenotypic clusters (*P* = 0.013). APC2 had many of the hallmarks of allergic asthma. APC2 also had the highest proportion of males (65.1% male), the youngest age of onset of asthma [3.4 (0.3) years], and the highest proportion of subjects currently using inhaled steroids (ICS) (23.9%).

Asthma phenotypic cluster 3 (APC3) had the lowest mean BMI percentile [53.7 (4.5)] and the best outcomes for post-bronchodilator pulmonary function tests [e.g., post-bronchodilator FEV_1_, mean (SE): 109.2 (18.6)] (Table 2). This corresponded to the highest mean ACT score [23.1 (0.4)] and highest ITG composite score [84.2(72.9, 95.8)]. Furthermore, APC3 had the lowest proportion of subjects with positive allergen skin prick tests (25%). They also had the smallest proportion of patients currently using inhaled corticosteroids.

In our post hoc between-group analyses, APC1 and APC2 were similar to each other in terms of pulmonary function tests, questionnaire results, and other typical indicators of asthma severity than APC3 was to either APC1 or APC2. A Least significant difference (LSD) test showed several of the APC3 variables to be more different from APC1 and APC2 than APC1 and APC2 were from each other. For example, post-bronchodilator FEV_1_/FVC (% predicted) and change in FEV_1_ post-bronchodilator (% predicted) variables were statistically different between APC3 when compared to APC1 and APC2 (0.05 < *P* ≤ 0.005). The LSD test showed that between APC1 and APC2 post-bronchodilator FEV_1_/FVC (% predicted) and change in FEV_1_ post-bronchodilator (% predicted) were not statistically significant (*P* > 0.2).

As in Benton et al. [18], there was no significant correlation between NAEPP (National Asthma Education and Prevention Program) severity score and cluster membership assignment—the NAEPP score classifies asthma severity based on impairment and future risk. The distribution of NAEPP severity scores was heterogeneous within and between the groups.

### The taxonomic composition of the nasal microbiome

We collected 205 nasal washes from all participants during one or two consecutive visits and sequenced the variable region V4 of the 16S rRNA using the Illumina MiSeq platform. Longitudinal differences between nasal microbiomes sampled twice in this study have been described in a previous publication by our group [29]. Here, we focus on the comparison of asthma phenotypes. A total of 6,386,235 sequences ranging from 530 to 160,718 sequences per sample (mean = 25,932; median = 31,152.4) were obtained after quality control analyses. From these data, we identified a total of 8034 OTUs (Additional file 3: Table S2; OTU taxa).

**Fig. 1:**
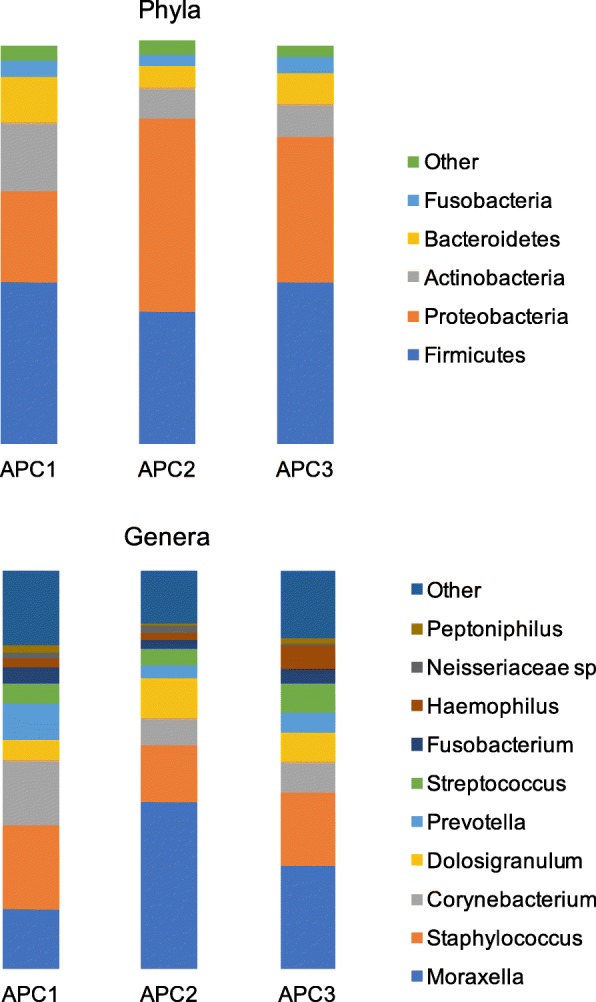
Microbial profiles (mean relative proportions) of most abundant (> 1%) phyla and genera in the nasal microbiomes of children and adolescents belonging to three different asthma phenotypic clusters (APCs)

The nasal microbiomes across all 205 samples included sequences that corresponded to five dominant (> 1%) Phyla: Firmicutes (37.8), Proteobacteria (36.3%), Actinobacteria (11.1%), Bacteroidetes (8.2%), and Fusobacteria (3.5%) (Fig. 1). Those Phyla comprised 10 dominant (> 1%) genera: *Moraxella* (28.3%), *Staphylococcus* (17.8%), *Corynebacterium* (10.1%), *Dolosigranulum* (7.7%), *Prevotella* (5.5%), *Streptococcus* (5.5%), *Fusobacterium* (3.3%), *Haemophilus* (3.2%), Neisseriaceae sp. (1.4%), and *Peptoniphilus* (1.2%) (Fig. 1). All the other detected genera accounted for < 1% of the total 16S rRNA sequences each. Each of the 205 nasal microbiomes contained 3 to 10 (mean = 7.8 genera) of the dominant bacterial genera. All these genera are commonly found in the nose of infants and adults with and without asthma, although in different proportions [24–29, 33, 34, 36, 60–67]. Nonetheless, previous studies [25, 68] have also revealed that the nose includes microenvironments containing microbiotas with different diversity and structure and that nasal washes may only capture part of that diversity. Hence, the diversity and complexity of the nasal microbiome is probably larger than what we show here.

A variable number of OTUs from these 10 dominant genera were included in the NP core microbiome, which potentially comprises the stable and consistent members and associations from the whole community [69, 70]. The least stringent definition of the core (presence in at least 50% of the samples) identified 44 OTUs of commensal and pathogenic bacteria; while a more stringent definition (presence in at least 95% of the samples) included four OTUs of the following genera: *Moraxella*, *Staphylococcus*, *Streptococcus*, and *Haemophilus*. Pathogenic representatives from these four genera have been consistently associated to asthma [23, 24, 27, 28, 30, 31, 34, 36, 60]; hence, these four OTUs may represent fingerprints or biological markers of the NP microbiome in asthmatic children. Future metagenomic studies will need to confirm their consistency across asthmatic cohorts, nasal microenvironments [25, 68] and specificity (i.e., dominant in asthmatics and absent in healthy controls).

### The nasal microbiome of asthmatic children and adolescents varies across phenotypic clusters

Microbial abundances of all the five dominant (< 1%) bacterial phyla varied across the three asthma phenotypic clusters (Table 3 and Fig. 1). APC1 showed the highest abundance of Actinobacteria and Bacteroidetes, high abundances of Firmicutes and Fusobacteria, and the lowest abundance of Proteobacteria; APC2 showed the highest abundance of Proteobacteria and the lowest abundance of the other four phyla; and APC3 showed intermediate proportions of Proteobacteria, Actinobacteria, and Bacteroidetes and high abundances of Firmicutes and Fusobacteria (as APC1). Our LME analyses showed significant differences in the mean relative proportions of Proteobacteria (*P* = 0.0005), Actinobacteria (*P* = 0.001), and Bacteroidetes (*P* = 0.0491) across the three asthma phenotypic clusters.

**Table 3:** Mean alpha-diversity indices and mean relative proportions of dominant phyla and genera (> 1%) in decreasing order of abundance for ALL samples and across three asthma phenotypic clusters (APC1, APC2, and APC3) in pediatric asthma

Taxon	ALL	APC1	APC2	APC3	*F*	DF	*P*(>*F*)
Alpha-diversity							
ACE	225.9	227.3	217.9	234.6	0.8	151	0.4710
PD	21.5	21.5	21.5	21.6	0.02	155	0.9787
Shannon	1.82	2.02	1.59	1.88	3.1	141	0.0445
Beta-diversity							
UniFrac-w	–	–	–	–	5.2	2	0.0001
UniFrac-unw	–	–	–	–	1.1	2	0.1156
Bray-Curtis	–	–	–	–	3.6	2	0.0001
Jaccard	–	–	–	–	2.7	2	0.0002
Phyla							
Firmicutes	37.8	40.4	33.3	40.7	0.6	139	0.5548
Proteobacteria	36.3	22.8	48.4	36.2	5.4	140	0.0056
Actinobacteria	11.1	17.4	7.5	8.4	4.7	136	0.0105
Bacteroidetes	8.2	11.5	5.7	7.7	3.1	153	0.0491
Fusobacteria	3.5	4.2	2.5	4.1	1.4	126	0.2523
Genus							
*Moraxella*	28.3	15.2	41.7	25.8	6.1	141	0.0029
*Staphylococcus*	17.8	20.9	14.5	18.5	0.9	142	0.4013
*Corynebacterium*	10.1	16.2	6.7	7.5	4.7	136	0.0110
*Dolosigranulum*	7.7	5.3	10.0	7.4	3.0	146	0.0484
*Prevotella*	5.5	8.8	3.0	5.1	3.8	155	0.0242
*Streptococcus*	5.5	5.2	4.3	7.4	1.1	150	0.3518
*Fusobacterium*	3.3	3.9	2.4	3.7	1.1	126	0.3251
*Haemophilus*	3.2	2.4	1.9	5.6	1.6	113	0.2056
Neisseriaceae sp	1.4	1.6	1.7	0.7	1.3	156	0.2643
*Peptoniphilus*	1.2	1.8	0.6	1.2	0.9	156	0.4201

Linear mixed-effects (LME) models results are shown for alpha-diversity indices and taxa abundances, while permutational multivariate analysis of variance (adonis) results are shown for beta-diversity indices. The significance of LME models was estimated using ANOVA of type III with Satterthwaite approximation for degrees of freedom. For each test, we report the relevant *F* statistic (*F*), degrees of freedom (DF), and significance (*P*(> *F*))

Microbial profiles of some of the most abundant bacterial genera (Table 3 and Fig. 1) also varied across asthma phenotypic clusters. APC1 showed the highest abundance of *Corynebacterium* and *Prevotella* and the lowest abundance of *Moraxella* and *Dolosigranulum*; APC2 showed the highest abundance of *Moraxella* and the lowest abundance of *Corynebacterium*, *Staphylococcus*, and *Prevotella*; while APC3 showed an intermediate abundance of those five genera. Our LME analyses showed significant associations with asthma phenotypic cluster for *Moraxella* (*P* = 0.0003), *Corynebacterium* (*P* = 0.0014), *Dolosigranulum* (*P* = 0.0484), and *Prevotella* (*P* = 0.0256). All these significant associations above between phyla and genera and asthma phenotypic clusters were confirmed by the Wald test with Cook’ s distance correction for outliers (0.05 ≤ *P* ≤ 0.0001) while accounting for preterm birth.

Alpha-diversity index (Shannon, ACE, and PD) varied across asthma phenotypic clusters (Additional file 4: Figure S2), but only Shannon estimates, which showed less diversity for APC2, were significantly different (*P* = 0.024) in our LME analyses (Table 3).

PCoAs of UniFrac (unweighted and weighted), Bray-Curtis, and Jaccard distances showed partial segregation of the microbiotas from each asthma phenotype (Additional file 5: Figure S3). Our adonis analyses detected significant differences (*P* ≤ 0.0002) in beta-diversity among phenotypes for all of the four distances but UniFrac unweighted (*P* = 0.1172). Mantel (*r* = 0.54, *P* = 0.038) and Procrustes tests showed a significant correlation between asthma phenotypic diversity and nasal microbial diversity (sum of squares M^2^ = 0.29, *P* = 0.001).

**Fig. 2:**
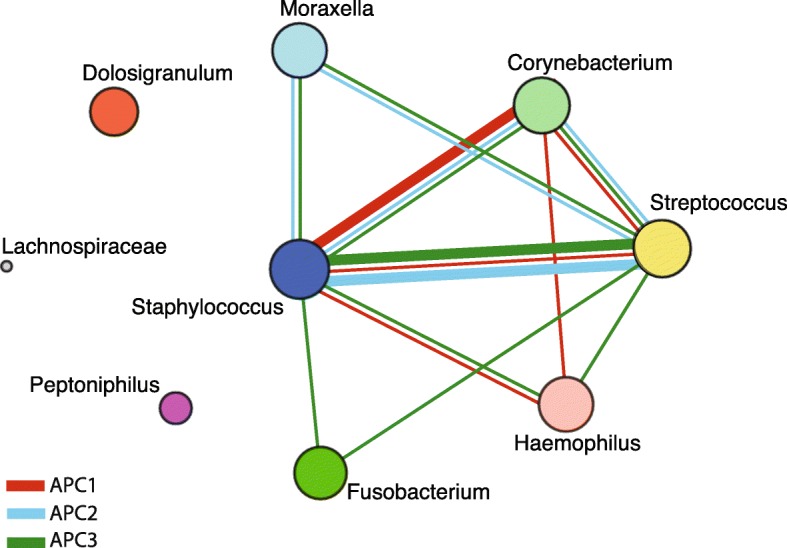
Network analyses of microbial co-occurrences in the nasal microbiomes of children and adolescents belonging to three different asthma phenotypic clusters (APCs). Different network edge colors were used for each phenotype. Thin edges correspond to probabilities of 0.90, while thick edges correspond to probabilities of 0.95. Only bacterial genera involved in a network are displayed

Genus-based microbial interaction networks varied across the three asthma phenotypic clusters (Fig. 2). Only co-occurrence (positive) relationships were detected under our parameter settings. APC1 (red) and APC2 (cyan) involved four genera and five edges, while APC3 (green) involved 6 genera and 9 edges at probability = 0.9. All phenotype networks involved two genera and one edge at probability = 0.95. There was partial overlap among all networks, with genera *Staphylococcus*, *Streptococcus*, and *Corynebacterium* being involved in all of them.

Preterm birth was significantly associated to variation in alpha-diversity (Shannon; *P* = 0.005), all beta-diversity indices (*P* ≤ 0.0008) except UniFrac unweighted distance, and the abundances of four taxa: Proteobacteria (*P* = 0.0017), Firmicutes (*P* = 0.0233), *Moraxella* (*P* = 0.0042), and *Staphylococcus* (*P* = 0.0167).

## Discussion

In this study, we investigated the composition and structure of bacterial communities inhabiting the nasal cavity of 163 asthmatic children and adolescents from the Washington, DC area. We first used clinical, physiological, and biochemical information to separate patients into three phenotypic clusters corresponding to different phenotypes of asthma, and then used bacterial 16S rRNA sequences to compare their NP microbiotas across those three asthma phenotypic clusters.

### Pediatric asthma comprises different phenotypic clusters

Our cluster analysis of the AsthMaP-2 cohort identified three clusters corresponding to three pediatric asthma phenotypes. Clusters of similar proportion and characteristics were identified in a previous study of asthmatic children and adolescents, mainly African Americans, also from the DC area using the AsthMaP cohort [18]. AsthMaP-2 and AsthMaP are two consecutive longitudinal cohorts including different patients. AstMaP-2 is analyzed here for the first time using both clinical and microbiome data. The clinical variables used here to cluster by case overlapped with the 11 variables used to cluster the original AsthMaP cohort, but here, we used only 6 variables. Despite the use of fewer variables to cluster, the k-means cluster analysis still produced three clusters of similar clinical characteristics. Since in both cohorts we found three phenotypic clusters comprised of individuals of similar clinical characteristics, we may conclude that the studied asthma phenotypes are probably stable and reproducible.

Asthma phenotypic cluster 1 (APC1) had lower mean scores in terms of severity measures (ITGc score) and asthma control measures (ACT), as previously mentioned. This predominantly African American female cluster with a mean age of 12.1 (0.50) years has many of the hallmarks of patients at risk for refractory asthma. Benton et al. [18] previously established a phenotypic cluster of predominantly overweight or obese asthmatics in the AsthMaP cohort. This phenotypic cluster had a mean age of 13.8 (0.6) years. The potential re-appearance of this phenotypic cluster indicates a possible socio-biological context for this group’s disease process. Data from the Childhood Asthma Management Program (CAMP) also suggests that the onset of puberty in both sexes represents a turning point in the disease progression for both females and males. Female symptoms worsen during puberty while male symptoms improve in late puberty [71]. This, in conjunction with our cluster analysis, suggests there may be an association between sex hormones and the pathogenesis of asthma.

APC2 was predominantly male with a preponderance of positive allergic asthma measures (e.g., blood eosinophil %, serum IgE) and the lowest mean age of 9.1 (0.3) years. The IgE antibody production in asthma patients mediates hypersensitivity reactions; it binds to receptors in mast cells and basophils and triggers the release of mediators. APC2 high mean IgE values indicate atopy in this phenotypic cluster.

APC3 also responded to bronchodilator use measured by the pulmonary function tests (e.g., post-bronchodilator FEV_1_) and asthma control questionnaires. APC3 was also predominantly male and had the lowest mean BMI percentile of 53.7 (0.6). Benton et al. [18] also introduced a similar phenotypic cluster (81% male) with a mean BMI percentile of 51 (3.2) in the AsthMaP cohort. This cluster also had the highest mean ITGc score of the three clusters. Another study of a cohort of African American and Hispanic children in New York City has demonstrated an inverse correlation between asthma risk and extreme BMI percentiles (underweight and overweight, 95th percentile) in young males [72]. This phenotype of low-BMI males with asthma appears in both the AsthMaP and AsthMaP-2 cohorts for the males with the lowest BMI percentiles.

### The nose is a reservoir for opportunistic pathogens

The nose is the major ecological niche for potential pathogens that cause lower respiratory infections such as asthma [23, 24, 27, 28, 30, 31, 34, 36, 60]. All core microbiome genera (Fig. 1) we detected in the nose (*Moraxella*, *Staphylococcus*, *Streptococcus*, and *Haemophilus*) include opportunistic pathogenic species of the airways [73, 74]. Other population-based microbiome studies of the lower and upper respiratory airways (excluding the nose and nasopharynx) have also shown enrichment of these genera in asthmatic infants, children, or adults [20, 34, 75–77]. Thus, our results confirm in children and adolescents the potential role of the nose as a reservoir of pathogens for other sections of the respiratory tract [23, 29, 34].

### The nasal microbiome changes across pediatric asthma phenotypes and preterm birth

The relationship between airway microbiology and asthma phenotypes is still poorly understood [37–39]. Here, we investigated if unbiased phenotypic clusters of pediatric asthma and clinical, physiological, and biochemical characteristics were associated with nasal bacterial diversity. Microbial profiles of some of the most abundant bacterial phyla and pathogenic genera associated with asthma (e.g., Proteobacteria and *Moraxella*) varied significantly (LME, *P* < 0.05) across pediatric asthma phenotypic clusters. Similarly, both alpha-diversity (Shannon index) and beta-diversity (UniFrac weighted, Bray-Curtis, and Jaccard distances) estimates revealed significant differences (Table 3; LME, *P* < 0.05) in the composition (abundance and evenness) and structure, respectively, of the microbial communities of the three asthma phenotypes. Additionally, microbial co-occurrences (as indicated by our network analysis) among dominant bacterial members also varied across asthma phenotypic clusters (Fig. 2). This likely reflects different symbiotic interactions between pathogenic and commensal bacteria in the nose as seen in other respiratory diseases [65, 78]. Nonetheless, taxa co-occurrences as described here, do not necessarily reflect functional relationships. Further studies, focusing on the functional capabilities these taxa display within the nose (e.g., via RNASeq analyses, see [24]) are needed to determine if these bacteria truly contribute to symbiosis during asthma.

By combining microbiome and clinical information from the same cohort of individuals, we were able to distinguish different disease variants of pediatric asthma with potentially different etiologies and pathophysiologies. No other study we are aware of has studied the relationships between pediatric asthma phenotypes and airway microbiomes, but a few studies have investigated microbiome-phenotype associations in adult asthma. Taylor et al. [37] found that participants with four different inflammatory asthma phenotypes (as indicated by relative proportions of neutrophils and eosinophils) also showed differences in composition and structure for both pathogens and commensal bacteria. The study also revealed differences in the abundance of opportunistic pathogens, like *Moraxella*, among inflammatory phenotypes. In our study, *Moraxella* abundance also varied significantly (LME; *F* = 5.6; DF = 119; *P* = 0.0194) in relation to eosinophil proportions across pediatric phenotypes—we did not collect neutrophil information. Future studies will need to assess if microbial profiles seen in adults with different inflammatory asthma phenotypes mimic (or develop from) those seen in asthmatic children and adolescents.

Another study by Zhang et al. [38] also revealed marked differences in the distribution of bacterial phyla (Proteobacteria and Firmicutes) and genera (*Streptococcus* and *Prevotella*) between two phenotypes of asthma severity (severe and non-severe) in adults. They did not observe differences in microbial composition (alpha-diversity) between phenotypes, but the opposite was true for microbial community structure (beta-diversity). In our study, asthma severity was classified as mild, moderate, intermediate, and severe, and it was not significantly associated (*P* > 0.07) with microbial diversity or taxa abundances. Several factors may explain the different outcomes between our results and Zhang et al.’s study; firstly, the studied cohorts have different characteristics, including patient age, ethnicity, country of residence, and treatment; secondly, the analyzed sample types were different—we used nasal washes while Zhang et al. used induced sputum; thirdly, the applied asthma severity classification systems are different—we used the National Asthma Education and Prevention Program (NAEPP) [79], while Zhang et al. used the Severe Asthma Protocol [80]. The microbiome similarity seen in our study across asthma severity types is supported by our cluster analysis and agrees with a previous clinical study of 154 children in the AsthMaP project (Washington, DC) [18], which also did not find significant differences in NAEPP severity scores and cluster membership assignment. Our study, hence, confirms previous results [18] and suggests that a clustering method like the one used here coupled with microbial profiles could be used as a new strategy to group asthmatic children and adolescents based on their asthma phenotype. Future work is needed to investigate the efficacy of current and new treatment options for these asthma phenotypes.

Preterm birth was significantly associated with variation in microbiome diversity and abundance of several microbial taxa. Individuals born prematurely undergo dramatically different early-life exposures in the neonatal intensive care unit (NICU). Prematurity-related challenges include nosocomial pathogens, supplemental oxygen, mechanical respiratory support, broad-spectrum antimicrobials, and deprivation of the normal intrauterine environment. Previous studies have shown that the airway microbiome may also play a role in post-natal problems faced by premature infants [73, 81]. A recent study has shown that the NP microbiota of premature infants is altered relative to that of infants born at term and that those changes persist during at least early childhood (6 months to 2 years) [82]. Microbial dysbiosis may play an important role in modulating airway inflammatory and immune responses [83]. Indeed, previous studies have established that the early nasal and nasopharyngeal microbial composition correlates with individual frequency and severity of upper and lower respiratory infections as well as subsequent risk of developing asthma [27, 33, 84–86]. Our results suggest that nasal microbiome changes acquired by preterm neonates may persist into adolescence. Future longitudinal studies will be needed to further investigate the interplay between preterm birth, nasal microbiota, and the development of airway immune responses against respiratory pathogens in early life.

### Limitations

Metataxonomic studies like ours suffer from the inherent limitations of collecting sequence data from a single partial gene target (16S rRNA) [87]. First, there is no validation of the composition and structure of the microbiotas using an alternative molecular marker. There are well-known issues with bias in PCR amplification early in the PCR reaction that can impact microbial compositional assessment. The single partial gene approach has also limited resolution at the species and sometimes even genus level for taxonomic assignment. Nevertheless, the composition of the nasal microbiomes in this study is similar to those described in previous microbial studies of the nose and nasopharynx in asthmatics using the same cohort but different individuals [25, 29, 60] or different cohorts [27, 33, 34]. Second, the relevance of detecting organisms associated with specific phenotypes of clinical variables is unknown—do the taxa that differentiate asthma phenotypic clusters engage in distinct interactions with the host or induce features consistent with the phenotypic features of the cluster in which they are enriched? Previous research by our group [24, 26] has used dual transcriptomics (RNAseq) to investigate host-microbe interactions during asthma. Metatranscriptomic insights coupled with longitudinal sampling (as oppose to the cross-sectional sampling design using here) can help to clarify whether specific microbes are drivers or bystanders in asthmatic patients. Our future microbiome research will address this issue. Lastly, we did not include healthy control samples in our study; hence, we cannot test how bacterial profiles in asthmatics compare to healthy children of similar age. Multiple studies, however (including ours), have already established that the nasal microbiomes of asthmatic and healthy individuals differ across ages in infants, children, and adults [20, 21, 29, 34, 77]. The focus of our study was different since we aimed to determine if asthmatic children belonging to different asthma phenotypes also have different microbiomes. Our results demonstrate this difference in a rather large cohort; however, including control samples would help to further validate if relative microbial enrichments are exclusive to children with asthma or are also detected in upper airways of healthy children or non-asthmatic individuals with a relevant history of nasal allergies or frequent respiratory infections [29, 77, 88].

## Conclusions

Microbiome information has been consistently neglected in the study of asthma phenotypes. Our study identifies significant differences in the composition and structure of the nasal microbiotas of children and adolescents across asthma phenotypic clusters. Microbial profiles (e.g., *Moraxella* and *Corynebacterium*) coupled with clinical, physiological, and biochemical factors (e.g., ITG and ACT scores) revealed different disease variants of pediatric asthma. This information could ultimately inform our understanding of asthma pathophysiology, validate (and further refine) current asthma classifications, improve current prognostic markers (i.e., asthma biomarkers) of disease [18, 37], and custom-fit treatment options for precision medicine. This study represents a step forward towards that ultimate goal.

## Additional files

Additional file 1: **Table S1.** Clinical and demographic characteristics of the cohort analyzed in this study. (XLSX 70 kb)

Additional file 2: **Figure S1.** Heatmap of six clinical variables (ACT score, age, BMI percentile, sex, ITGc and blood eosinophil %) showing three asthma phenotypic clusters (APCs). (PDF 903 kb)

Additional file 3: **Table S2.** Number of clean sequences per OTU and sample and corresponding taxonomic identification up to the genus level. (XLSX 4706 kb)

Additional file 4: **Figure S2.** Box plots of Shannon, ACE and phylogenetic alpha-diversity of microbiotas from children and adolescents belonging to three asthma phenotypic clusters (APCs). (PDF 1885 kb)

Additional file 5: **Figure S3.** Principal coordinates analyses of unweighted and weighted UniFrac, Bray-Curtis and Jaccard distances among microbiotas from children and adolescents belonging to three asthma phenotypic clusters (APCs). (PDF 1160 kb)
