# Supplementary figures and images for: Pediatric asthma comprises different phenotypic clusters with unique nasal microbiotas
Source: Microbiome. 2018 Oct 4;6:179. doi: 10.1186/s40168-018-0564-7 (PMC6172741; doi:10.1186/s40168-018-0564-7)

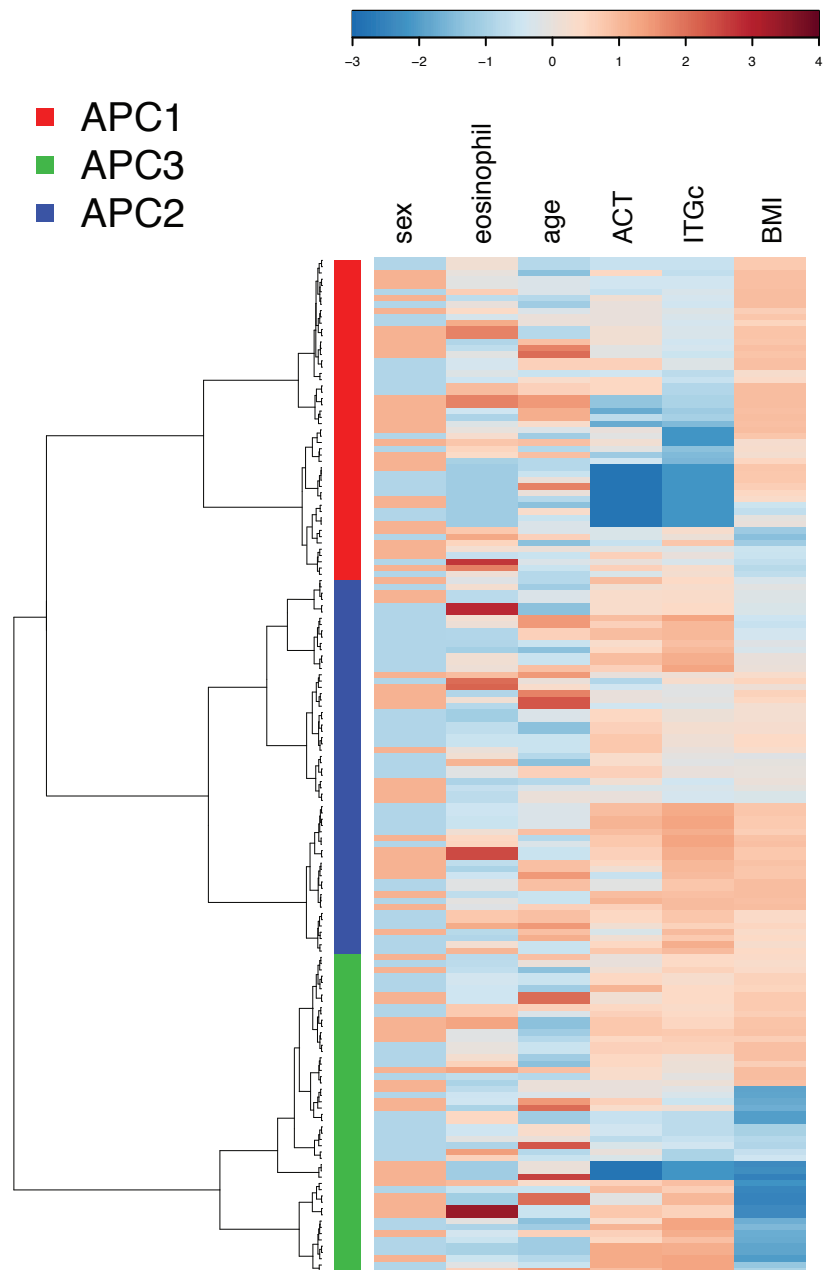

Supplement: Supplementary file 2 — Figure S1. Heatmap of six clinical variables (ACT score, age, BMI percentile, sex, ITGc and blood eosinophil %) showing three asthma phenotypic clusters (APCs). (PDF 903 kb) [file 40168_2018_564_MOESM2_ESM.pdf]

Shannon diversity

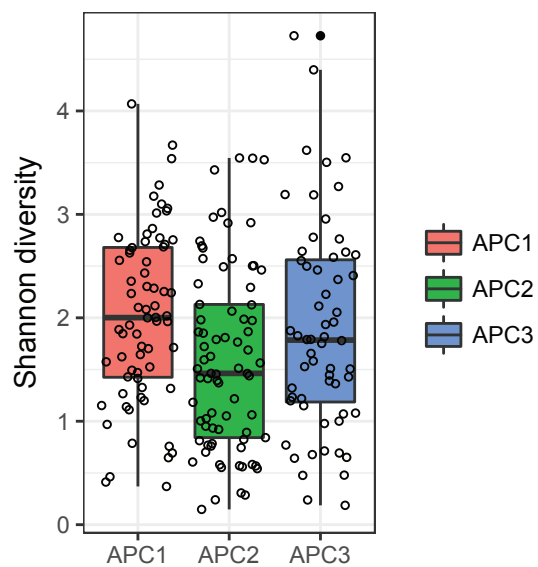

Phylogenetic diversity

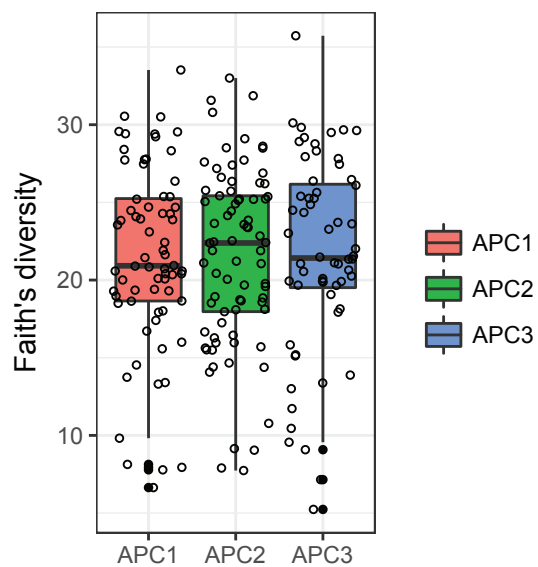

ACE diversity

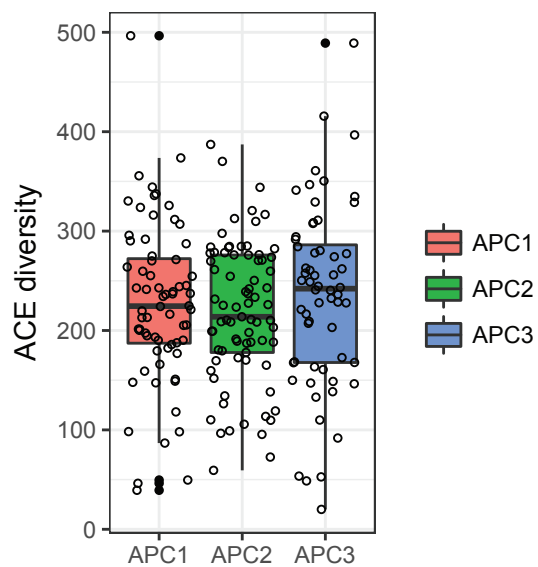

Supplement: Supplementary file 4 — Figure S2. Box plots of Shannon, ACE and phylogenetic alpha-diversity of microbiotas from children and adolescents belonging to three asthma phenotypic clusters (APCs). (PDF 1885 kb) [file 40168_2018_564_MOESM4_ESM.pdf]

PCoA Unweighed UNIFRAC

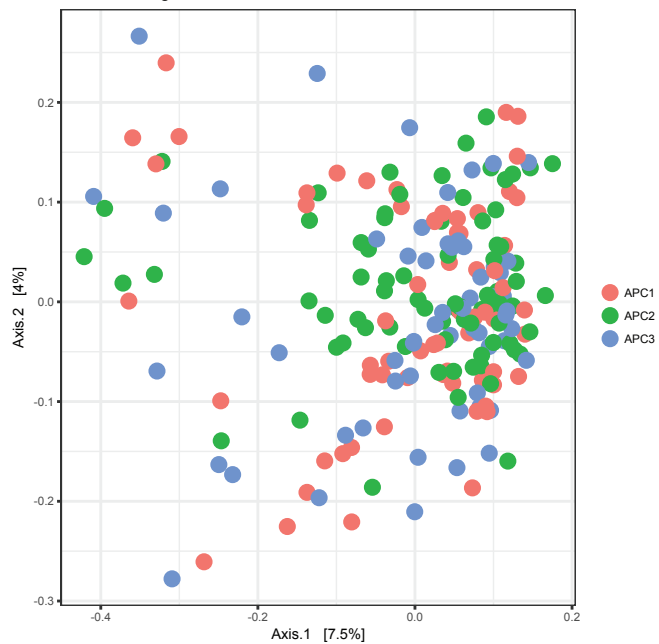

PCoA Weighed UNIFRAC

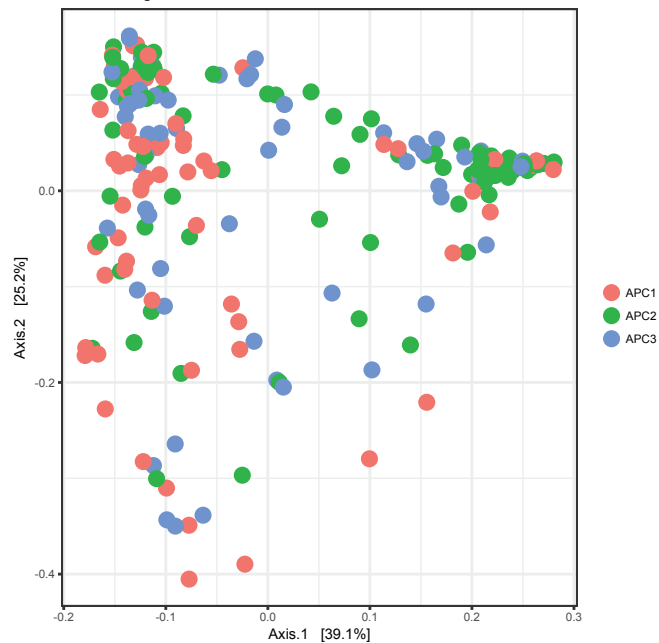

PCoA Bray-Curtis

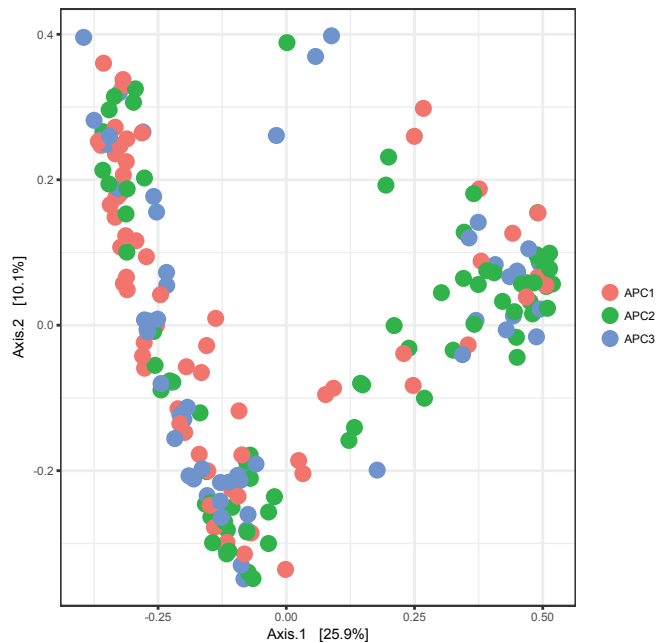

PCoA Jaccard

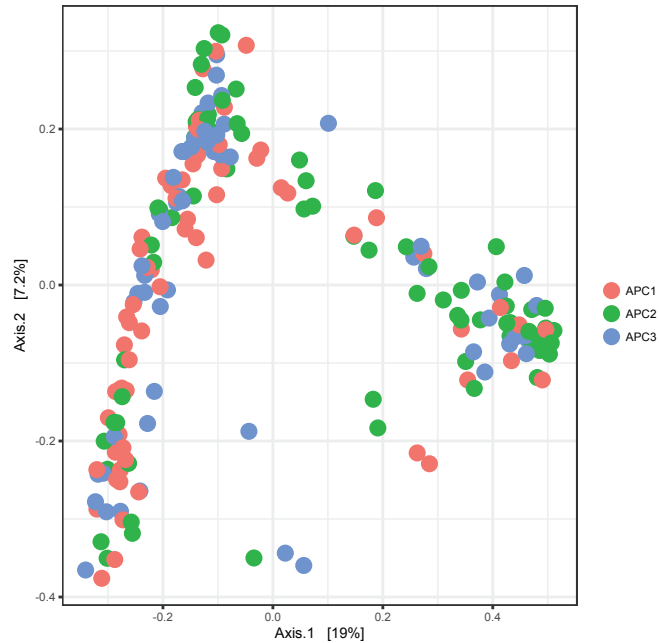

Supplement: Supplementary file 5 — Figure S3. Principal coordinates analyses of unweighted and weighted UniFrac, Bray-Curtis and Jaccard distances among microbiotas from children and adolescents belonging to three asthma phenotypic clusters (APCs). (PDF 1160 kb) [file 40168_2018_564_MOESM5_ESM.pdf]
